# Prospective memory mediated by interoceptive accuracy: a psychophysiological approach

**DOI:** 10.1098/rstb.2016.0005

**Published:** 2016-11-19

**Authors:** Satoshi Umeda, Saiko Tochizawa, Midori Shibata, Yuri Terasawa

**Affiliations:** 1 Department of Psychology, Keio University, Tokyo 108-8345, Japan; 2 Keio Advanced Research Center, Keio University, Tokyo 108-8345, Japan

**Keywords:** prospective memory, interoception, cardiac reactivity, autonomic nervous activity

## Abstract

Previous studies on prospective memory (PM), defined as memory for future intentions, suggest that psychological stress enhances successful PM retrieval. However, the mechanisms underlying this notion remain poorly understood. We hypothesized that PM retrieval is achieved through interaction with autonomic nervous activity, which is mediated by the individual accuracy of interoceptive awareness, as measured by the heartbeat detection task. In this study, the relationship between cardiac reactivity and retrieval of delayed intentions was evaluated using the event-based PM task. Participants were required to detect PM target letters while engaged in an ongoing 2-back working memory task. The results demonstrated that individuals with higher PM task performance had a greater increase in heart rate on PM target presentation. Also, higher interoceptive perceivers showed better PM task performance. This pattern was not observed for working memory task performance. These findings suggest that cardiac afferent signals enhance PM retrieval, which is mediated by individual levels of interoceptive accuracy.

This article is part of the themed issue ‘Interoception beyond homeostasis: affect, cognition and mental health’.

## 1. Introduction

Prospective memory (PM) refers to an individual's ability to remember intentions or to plan or perform an action at some point in the future. PM is pervasive in everyday life, for example, remembering to buy daily necessities or take medication at the appropriate time. It is divided broadly into two types, event-related PM and time-based PM. In event-related PM, the action (e.g. posting a letter) is cued when the target event occurs (e.g. seeing the mailbox). Individuals attempt to remember the appropriate action that is associated with the target event. Time-based PM involves remembering to perform an action at a certain time (e.g. taking medication at 15.00 h). In general, opportunities to perform intended tasks occur in the midst of other ongoing activities. Thus, completion of tasks stored in PM often requires multitasking. Several previous studies have used a dual PM task paradigm, in which the PM task was embedded in an ongoing task, to tackle issues related to the mechanisms of PM. This has enabled the execution of delayed intended activities to be evaluated in laboratory-based settings [1]. In the event-related PM task, participants are instructed to perform a prospective action only when the specified event occurs or the PM target is presented (e.g. press the ‘F1’ key when you see animal names) [2].

Previous studies on PM have described a variety of approaches, including cognitive behavioural, developmental, gerontological, neuropsychological, neuroimaging and psychophysiological analyses. Among these, conventional behavioural studies have led to the development of experimental methods and theories [3]. In particular, during the last decade, novel theories and models have been proposed to better understand the PM retrieval process. Debate is ongoing with respect to two plausible explanations: the preparatory attentional and memory processes theory [4–8] and the multiprocess theory [9]. These theories differ with respect to the automatic cognitive components involved in PM retrieval (see the Discussion section). The neural substrates of PM have been explored using neuropsychological and neuroimaging techniques. These studies suggest that the dorsolateral prefrontal area (Brodmann area 9/46), the anterior prefrontal area (Brodmann area 10) and several components of the medial temporal area contribute to PM retrieval [10–15]. In addition, research using event-related brain potentials (ERPs) has investigated the temporal dynamics of the mental processes underpinning PM [16–19].

From the psychophysiological perspective, a considerable number of studies have outlined the effects of autonomic activities on various cognitive functions, including attention or target detection, which share the PM component [20–23]. Previous studies indicate that psychological stress enhances memory retrieval [24,25], and that psychologically significant stimuli elicit an increase in heart rate [22,26]. According to the implications of the Zeigarnik effect, individuals tend to experience psychological stress when sustaining a delayed intention [25]. If a person is unsuccessful in accomplishing the goal, the target thought can become more compelling [27]. More frequent self-monitoring triggered by the excessive thought and newly formed intention to suppress the thought can both exacerbate stress, which elevates sympathetic nerve activity. Although only a few studies have focused on the relationship between autonomic nervous activities and PM retrieval, it has garnered increasing attention during the last decade. Studies using skin conductance responses (SCR) as a measure of sympathetic nervous activity reported that detecting the PM target elicited responses [28,29]. Since the somatic marker hypothesis was proposed, many studies have reported a strong correlation between emotional reactions and autonomic nervous activity [30,31]. Similar to emotional reactions, it is reasonable to speculate that the initiation of memory retrieval required for successful PM performance is triggered by afferent signals based on the saliency detection by the presented stimulus. Some previous behavioural studies on metamemory suggested that the rapid preliminary feeling-of-knowing judgement precedes initiation of the actual memory retrieval process [32,33]. Although it remains unclear what elicits the feeling of familiarity, it is possible that autonomic body signals contribute to rapid and implicit PM retrieval, which is mediated by interoceptive accuracy.

Previous studies with the perspective of individual differences have investigated the relationship between PM performance and personality [34–37]. A positive correlation was found between PM performance and conscientiousness, perfectionism and neuroticism [35]. In terms of the dimensions of mood disorders, state anxiety was negatively correlated with the PM component (remembering to remember) in PM [38]. However, the factors that influence these correlations remain unresolved. It may be the case that some autonomic body responses have interactive roles. To address this question, the effects of autonomic bodily responses on PM performance have been evaluated by focusing on interoceptive accuracy, the ability to perceive afferent information that arises within the body [39–41]. Some studies indicate that interoceptive accuracy, as measured by performance on a heartbeat detection task (HDT), reflects individual emotional sensitivity and personality [42,43]. Individuals with high sensitivity to their own heartbeat tended to exhibit higher scores regarding anxiety and arousal levels in response to emotional stimuli [44], and to experience more salient emotions than those with low sensitivity [45,46]. Based on these findings, it was hypothesized that interoceptive accuracy is strongly correlated with PM performance.

In this study, cardiac reactivity was monitored as a measure of autonomic nervous activity, and the relationship between the change in heart rate and retrieval performance was evaluated in the event-based PM task. The main focus was to test the hypothesis that PM performance is influenced by cardiac activity and mediated by an individual's interoceptive accuracy. Based on the predictions that cardiac afferent signals would enhance PM retrieval and increase the probability of successful PM performance, it was expected that individuals with higher PM performance would have a greater increase in heart rate in response to PM target presentations. To provide a perspective on individual differences, we also tested the hypothesis that increased cardiac activity on PM retrieval is related to interoceptive accuracy. In a previous study, higher interoceptive accuracy was associated with greater emotional sensitivity when viewing emotional facial micro-expressions [43]. This may generally imply that detecting subtle differences in presented stimuli depends on interoceptive accuracy according to an individual's bodily state. Therefore, individual interoceptive accuracy may influence the possibility of successful PM performance, which was tested by target detection in a modified event-based PM task.

## 2. Material and methods

### (a) Participants

Thirty-eight undergraduate students (30 females/8 males, aged 18–23 years, mean 19.9 years) participated in the study. Participants were assigned to the ‘known’ condition (*n* = 19) or the ‘unknown’ condition (*n* = 19) as described below. Two participants were excluded from psychophysiological data analysis due to incomplete cardiac data. None of the participants had a history of neurological, psychiatric or visual symptoms.

### (b) Design and procedure

#### (i) Main experiment

**Figure 1.**
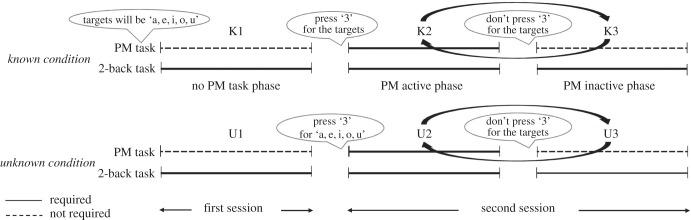
Task design. The main experiment included two concurrent tasks: an ongoing background task and a PM task. The ongoing task was the 2-back working memory task, in which participants were required to press key ‘1’ on the keyboard immediately in response to the letter that was identical to the letter seen two trials previously, and to press key ‘2’ when the criterion for pressing key ‘1’ was not met. This task used 10 consonant lower case letters (b, c, d, f, g, h, k, m, n, p). The PM task was the modified event-based PM task, in which participants were instructed to press key ‘3’ instead of pressing key ‘1’ or ‘2’ when they observed any of the five vowel letters ‘a, e, i, o, u’ (PM targets) during the ongoing working memory task. The first session was designed to examine the effect of knowledge about PM targets on cardiac reactivity, and the second session was designed to assess the behavioural and cardiac reactivity induced by presentation of PM targets.

The main experiment included two concurrent tasks: an ongoing background task and a PM task (figure 1). The ongoing task was the 2-back working memory task, in which participants were required to press key ‘1’ on the keyboard immediately in response to the letter that was identical to the letter seen two trials previously, and to press key ‘2’ when the criterion for pressing key ‘1’ was not met. In a trial, each letter was displayed for 500 ms followed by a fixation cross for 2500 ms. This task used 10 consonant lower case letters (b, c, d, f, g, h, k, m, n, p) that were displayed in black over the white background on the screen, viewed from a distance of approximately 50 cm, and subtended at a visual angle of 1.53° in width and 0.48° in height. Before starting the task, all participants conducted 18 practice trials to ensure that they understood the instructions.

The PM task was the modified event-based PM task, in which participants were instructed to press key ‘3’ instead of pressing key ‘1’ or ‘2’ when they observed any of the five vowel letters ‘a, e, i, o, u’ (PM targets) during the ongoing working memory task. However, these instructions for the PM task were not given to all participants at the same time, and the timing of instructions was dependent on the condition. Throughout the main experiment, trials of the occurrence of the PM target (pressing ‘3’ was correct) was 10%, trials with identical working memory (pressing ‘1’ was correct) was 30% and trials with non-identical working memory trials (pressing ‘2’ was correct) was 60% [47]. SuperLab 4.5 (Cedrus Corporation, USA: http://www.superlab.com/) was used to control stimulus presentations and response recordings. To obtain the responses, participants were asked to press keys ‘J’,‘K’ and ‘L’ on a keyboard to which stickers had been attached indicating ‘1’, ‘2’ and ‘3’, respectively.

The main experiment consisted of a total of 120 trials in two sessions. The first session was designed to examine the effect of knowledge about PM targets on cardiac reactivity. In the session (16 trials), participants were first asked to complete trials in the ‘No PM task phase’. Participants who were assigned to the ‘known’ condition were instructed to press ‘a certain key’ on the keyboard when they saw any of the vowel letters ‘a, e, i, o, u’ during the forthcoming second session (phase K1). They were also told that the certain key would be informed as key ‘3’ after performing some trials. By contrast, participants assigned to the ‘unknown’ condition were instructed to press a ‘certain key’ when they observed ‘certain letters’ during the forthcoming second session (phase U1). Therefore, when participants in the ‘known’ condition observed the PM targets, they would experience familiarity because they did not know the corresponding action to be performed. When the participants in the ‘unknown’ condition observed the PM targets, they were unfamiliar because they did not know the PM target or the corresponding action.

The second session was designed to assess the behavioural and cardiac reactivity induced by presentation of PM targets. It was started after the first session with the following instructions. Participants in the ‘known’ condition were requested to press key ‘3’ when they observed the PM targets (phase K2), whereas participants in the ‘unknown’ condition were instructed to press key ‘3’ when they observed any of the letters ‘a, i, u, e, o’ (phase U2) during an ongoing 2-back working memory task. They were also instructed that these were one-conditional phases (phase K2 or U2) and the other conditional phase would be prepared in which they were asked not to press key ‘3’ when any PM target was observed (phase K3 in the known condition and U3 in the unknown condition). The PM active phase (phase K2 or U2) and the PM inactive phase (phase K3 or U3) were switched with brief instructions for the participants about the session change, without any detailed instructions including the PM targets and the corresponding actions. Overall, phases K2 and U2, and phases K3 and U3 were treated as equivalent. Taking into account the possibility of adaptation or strategic responses to the task, we randomized the number of trials in each phase ranging from 8 to 16. The second session consisted of 104 trials. Participants were allowed to take a short break (about 1 min) after completing nearly half of all trials. To evaluate behavioural PM performance, we calculated the correct response ratio of the PM targets to the actual appearance of PM targets in the PM active phase (phase K2 and U2). Also, to evaluate working memory performance, we calculated the correct response ratio of the 2-back task in each phase in each condition. The design of the second session was more complex to minimize the possibility of behavioural habituation to the task and attenuation of cardiac responses.

#### (ii) Physiological recording

To measure cardiac reactivity, a pulse oximeter was attached to the left middle finger of each participant using the MP150 photoplethysmography system (PPG100C, BIOPAC Systems, USA: http://www.biopac.com/). Data acquisition and analyses were performed with AcqKnowledge 4.1 software (BIOPAC Systems, USA) on a Microsoft Windows PC. The spectral density of pulse data were sampled at 50 Hz. The inter-beat interval (IBI) was used to assess cardiac reactivity, according to the peak detection function implemented in the software. Events were excluded that deviated ±2 s.d. from the baseline cardiac beats in each participant, which was recorded for 3 min in a resting state before the experiment.

#### (iii) Assessment of interoceptive accuracy

To assess the interoceptive accuracy of participants, the HDT was used, which was originally developed by Schandry [44] and Ehlers & Breuer [48], and has been used in many studies [43,49–52]. Heartbeat was measured by pulse oximeter for specific periods of time (2 × 25 s, 2 × 35 s and 2 × 45 s). During the HDT, participants were asked to count the number of times they felt their own heartbeat during the measurement period, as well as during the main experiment period. They were instructed not to predict their heart rate. The pulse oximeter probe was gently placed on their fingertips to prevent participants from feeling the pressure of their pulse. They were instructed not to touch any part of their body during the task. Each trial began 3 s after the experimenter said ‘ready.’ HDT error rates were calculated based on the discrepancy between the number of reported and actual heartbeats during the measurement period. The formula used to calculate the HDT was based on that used by Ehlers & Breuer [48]: (∣actual heartbeats − reported heartbeats∣/actual heartbeats) × 100. Six HDT error rates were obtained for each participant, and the values were averaged to obtain the individual HDT error rate. Participants were then asked to report their normal heart rate (bpm) during daily life. If unable to report heartbeat, they were asked to make an estimate. The error rate of each reported heart rate was calculated using the above formula.

While participants were instructed not to predict their heart rate in the HDT, it is possible that they estimated the passage of time, thus affecting the HDT data and contaminating the measure of interoception. If this were the case, the HDT error rates should correlate with time estimation accuracy. However, Dunn *et al*. overcame this issue by demonstrating that HDT error rates do not correlate with time estimation accuracy [53]. We addressed this possibility by instructing participants to complete a time estimation task. In the task, they had to count the number of seconds during a specific period, and then the reported length was compared with the actual duration. We conducted six trials (2 × 23 s, 2 × 40 s and 2 × 56 s) and time estimation error rates were calculated in a manner similar to that of the HDT error rate. Each trial began 3 s after the experimenter said ‘ready,’ and participants reported their estimated duration immediately after each trial. Although data from the time estimation task were incomplete because of technical problems, most of the subjects in this study were enrolled in our previous study. These data confirmed that the HDT error rate did not correlate with time estimation accuracy (*r*_28_ = 0.35, n.s.) [43].

## 3. Results

### (a) Cardiac reactivity in the first session

**Figure 2.**
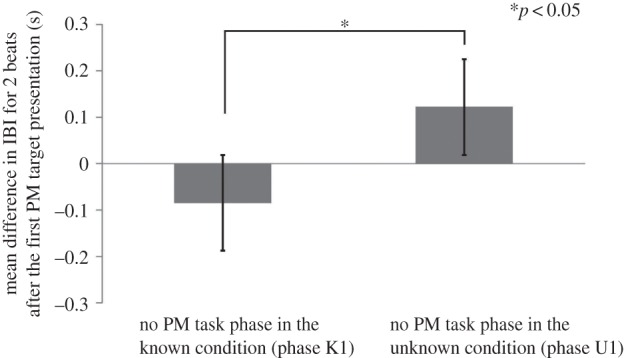
The event-related transition of the IBI from PM target presentations in the known (phase K1) and unknown condition (phase U1) in the first session, demonstrating an increase in heart rate in the known condition. This result indicated that higher cardiac reactivity was elicited by PM target presentations when the participants did not know the corresponding action to the PM targets.

To examine the effect of knowledge about PM targets on cardiac reactivity, we first calculated the event-related transition of the IBI from PM target presentations in the known (phase K1) and unknown conditions (phase U1). The beat-by-beat pattern of heart rate changes induced by stimulus presentation was carefully scrutinized, and then two heartbeats were selected as the most appropriate window for cardiac reflections to avoid contamination from the habituated responses during the session. The first IBI was selected to include initiation of the first PM target presentation (IBI-1), and the second IBI (IBI-2) was next to IBI-1. For each participant, the baseline IBI, which was prior to initiation of stimulus presentation, was calculated as zero (IBI-0), and determined as the relative average IBI value for two beats (IBI-1 and IBI-2). Thus, negative values correspond to an increase in heart rate. Using the values, one-way analysis of variance (ANOVA) was conducted for the known (phase K1) and unknown conditions (phase U1), which revealed a significant main effect of the condition (*F*_1,38_ = 4.39, *p* < 0.05; figure 2), demonstrating an increase in heart rate in the known condition. This result indicated that higher cardiac reactivity was elicited by PM target presentations when the participants did not know the corresponding action to the PM targets. The data showed the effect of PM target detection on cardiac reactivity, acting as supporting data for the interpretation of the second session.

### (b) Prospective memory and working memory performance in the second session

Behavioural performances on the PM task and the ongoing 2-back working memory task in the second session are shown in table 1. For PM measures, an omission error was defined as incorrectly pressing key ‘1’ or ‘2’ in response to the PM target, and a commission error was defined as incorrectly pressing key ‘3’ in response to the non-PM target. PM performance was tested by one-way ANOVA for the difference in correct response ratios in the PM task between the known (phase K2) and the unknown condition (phase U2). No significant main effect was found between the two conditions (*F*_1,36_ = 1.04, n.s.). To examine the differences in 2-back task performance in three phases for two conditions, two-way ANOVA was conducted with the phase (K1, K2, K3/U1, U2, U3) and the condition (known/unknown) as variables. No significant main effect was detected for the condition (*F*_1,36_ = 0.00, n.s.), but a significant main effect was found for the phase (*F*_2,72_ = 22.11, *p* < 0.001). No significant interaction was found between the two variables. Subsequent analysis revealed significant interactions in working memory performance between the no PM phase (K1, U1) and the PM active phase (K2, U2; *t*_72_ = 6.12, *p* < 0.001), and the PM active phase (K2, U2) and the PM inactive phase (K3, U3; *t*_72_ = 5.32, *p* < 0.001). These results indicated that working memory performance was reduced due to the concurrent PM task load. No significant difference was detected in PM performance and working memory performance between the known and unknown conditions. Thus, data for the two conditions were pooled in the following analyses.

**Table 1.** Behavioural performances on the PM task and the ongoing 2-back working memory task in the second session. Numbers in parentheses represent standard deviations (s.d.); PM, prospective memory.

phase	correct response ratio		error type	
	PM task	2-back task	omission	commission
known condition				
no PM (K1)	―	0.93 (0.06)	―	―
PM active (K2)	0.76 (0.20)	0.87 (0.07)	0.24 (0.20)	0.01 (0.03)
PM inactive (K3)	―	0.94 (0.05)	―	0.01 (0.01)
unknown condition				
no PM (U1)	―	0.94 (0.06)	―	―
PM active (U2)	0.82 (0.17)	0.87 (0.06)	0.18 (0.17)	0.01 (0.01)
PM inactive (U3)	―	0.92 (0.08)	―	0.00 (0.01)

### (c) Prospective memory, cardiac activities and interoceptive accuracy in the second session

**Figure 3.**
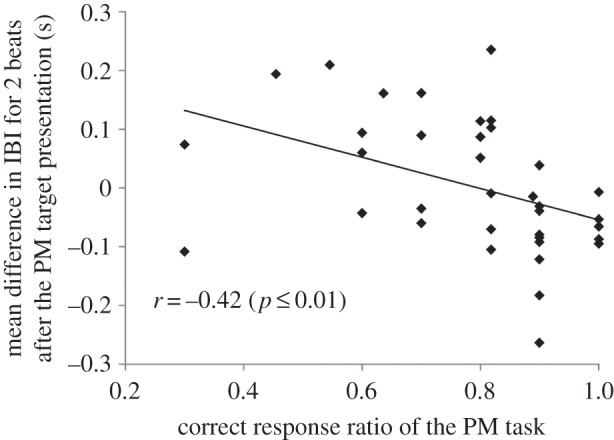
Correlation between the IBI values and the correct response ratios in the PM task. A significant correlation was found between the two variables, indicating that individuals with higher PM performance had a greater increase in heart rate after PM target presentation.

To examine the cardiac effects on PM performance, the mean IBI difference for two heartbeats from the baseline was calculated (as indicated above), and correlation analysis was conducted between the IBI values and their correct response ratios in the PM task (figure 3). A significant correlation was found between the two variables (*r*_34_ = −0.42, *p* ≤ 0.01), indicating that individuals with higher PM performance had a greater increase in heart rate at PM target presentation. By contrast, no significant correlation was detected between the IBI values and 2-back working memory task performance (*r*_34_ = −0.10, n.s.).

**Figure 4.**
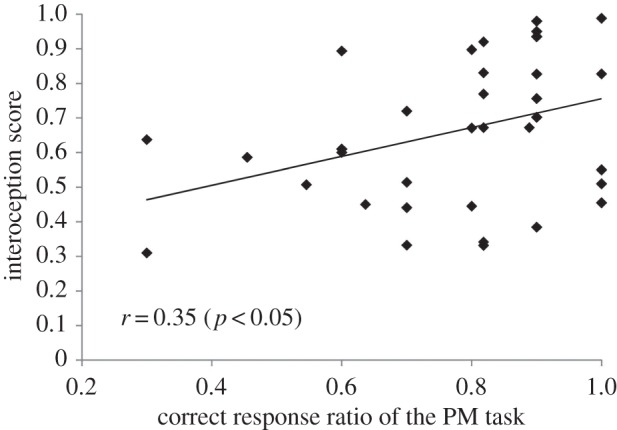
Correlation between interoceptive scores and the correct response ratios in the PM task. A significant correlation was found between the two variables, indicating that individuals with higher interoceptive perceiver results showed better PM task performance.

To verify the relationship between interoceptive accuracy (mean score = 0.67, s.d. = 0.22) and PM task performance, we conducted correlation analysis between interoceptive score, which was defined as one minus HDT error rate, and PM task performance (figure 4). A significant correlation was found between the two variables (*r*_34_ = 0.35, *p* < 0.05), indicating that individuals with higher interoceptive perceiver results showed better PM task performance. Contrary to the PM result, there was no significant correlation between interoceptive score and 2-back working memory task performance.

## 4. Discussion

In this study, we examined the relationship between cardiac activity and retrieval of a delayed intention in the event-based PM task. The main focus was to test the hypotheses that PM performance is mediated by cardiac activity and by individual interoceptive accuracy. Results of the second session supported both hypotheses. In terms of the first hypothesis, psychophysiological data showed that individuals with higher PM performance had a greater increase in heart rate on PM target presentation. This suggests that cardiac afferent signals may enhance PM retrieval and boost the probability of successful PM performance. Subsequently, the relationship between interoceptive accuracy and the cardiac enhancement effect on PM retrieval was investigated. Our previous study reported that more accurate interoceptive accuracy elicited more sensitive responses to the emotions of others when viewing emotional facial expressions [43]. This suggests that detecting subtle differences in presented stimuli depends on the interoceptive accuracy of an individual's bodily state. The PM targets in this study were not frequently displayed, and the subjective saliency of the PM targets may depend on interoceptive accuracy. Individuals with higher interoceptive accuracy, as measured by the HDT, showed better PM task performance. This may be evidence that the cardiac enhancement effect of PM performance is mediated by individual levels of interoceptive accuracy. Some previous studies on PM have proposed that ‘cue sensitivity’ is an influential factor in PM performance [54]. It is reasonable to suspect that cue sensitivity is highly associated with actual autonomic reactivity and interoceptive accuracy induced by the presented target cues.

Another key finding was that the correlation across interoceptive accuracy was only found for PM task performance, not for 2-back working memory performance. This implies that individual autonomic sympathetic activity and interoceptive accuracy have limited effects on memory performance. From the perspective of the underlying neural substrates, the concurrent 2-back task involves some processes that require working memory and cognitive control, which are realized by the executive function network, including the dorsolateral prefrontal cortex (DLPFC) and the anterior cingulate cortex (ACC). Although these components share the cognitive processes required for PM, cue sensitivity is likely to be the exclusive factor for successful PM retrieval, which may be implemented in the anterior prefrontal cortex [14,55]. Differences in neural substrates may affect the correlation between cardiac reactivity and interoceptive accuracy.

In the first session, the effect of knowledge about PM targets on cardiac reactivity was evaluated. Our findings demonstrated that higher cardiac reactivity was elicited by PM target presentation in the known condition than the unknown condition. This result indicated that the accelerated cardiac activity was elicited by PM target presentation, even if participants did not have any knowledge about the corresponding action associated with the PM targets. Some traditional studies indicate that psychological stress enhances memory retrieval [24,25], and also that psychologically significant stimuli elicit an increase in heart rate [22,26]. Clearly, the presented stimuli that were saliently detected as the forthcoming PM target had significant meanings that were distinct from other stimuli for the 2-back task. This may be interpreted as the readiness state for executing subsequently required actions following PM target detection. It may be regarded as a type of preparatory attentional process, which was observed as increased autonomic responses measured as accelerated cardiac reactivity. According to the two-component process model of PM, one component is defined as the process of noticing the stimulus, which is considered to be based on familiarity and cue sensitivity to the PM target, and the other component is defined as the process of a directed memory search based on the noticing process [1,28,56]. Our data suggest that cardiac reactivity was sufficiently triggered by only the former component, because no target was present for a directed memory search in the known condition. This finding is consistent with a previous psychophysiological study of PM using SCR [28], which showed that autonomic sympathetic activities were driven by the ‘noticing’ component of PM.

It is possible to argue that individuals with higher levels of stress exhibit higher autonomic reactivity. The Zeigarnik effect is likely to have an impact on PM performance because psychological stress is typically experienced when sustaining a delayed intention [25]. In the first session of this study, we experimentally manipulated the levels of pre-knowledge about future intentions. Our data suggest that more specific information about future intentions could elicit greater levels of psychophysiological stress. However, it remains unclear whether the change in cardiac activity reflects a stressed state caused by different instructions, rather than a reaction to PM target presentation. To clarify this point, a further experiment was conducted to compare cardiac reactivity elicited by PM target cues with that elicited by other cues in the 2-back working memory task. Increases in cardiac reactivity were unique to PM target cues (*F*_1, 19_ = 4.17, *p* ≤ 0.05). It is thus reasonable to support the notion that the accelerated cardiac reactivities were due to psychophysiological stress caused by the PM task component in this study.

As briefly discussed above, previous studies have argued on two distinct theoretical views of PM retrieval [9]. The preparatory attentional and memory processes theory assumes that after forming the intention of an event-based PM task, initial preparatory attentional processes are directed at considering environmental events as potential targets for PM intention [4,6,8]. Thus, PM retrieval only occurs when these preparatory attentional processes are engaged. On the other hand, the multiprocess theory assumes that the presence of a target event or cue spontaneously initiates retrieval of the PM intention from memory, even when no preparatory attentional processes are engaged [9,57]. According to the multiprocess theory of PM, cue detection is achieved through a relatively automatic process that is driven by differences in the degree of familiarity between the PM cues and other stimuli [56,58]. Although the present experiment was not designed to test the presence of a preparatory attentional process, the psychophysiological findings demonstrate that individuals with higher interoceptive perception had better PM task performance and individuals with higher PM performance had a more substantial increase in heart rate to PM target presentations, suggesting that changes in autonomic reactivity and interoceptive accuracy can mediate the relationship between detecting target cues and initiating the retrieval of PM intentions. Another concern which needs to be discussed is the distinction between focal and non-focal cue effects in PM [59]. The retrieval process of PM is considered to be relatively spontaneous in tasks with focal cues (e.g. respond to the word ‘tortoise’) as opposed to non-focal cues (e.g. respond to the syllable ‘tor’) [59,60]. Indeed, dementia-related decline was reported to be more robust in focal than non-focal PM [61]. In this study, we adopted the five vowel letters as PM target cues, which is considered to be focal. The interpretation of our results on the significant correlations between PM performance and heart rate change or interoceptive accuracy should be limited as findings using focal cues. Interestingly, a recent meta-analysis of neuroimaging studies of PM found that different brain areas were involved as a function of PM cue focality [11]. Further research will be needed to examine whether interoceptive accuracy has a similar role in non-focal cues, in which more attention-demanding monitoring strategies are required to support PM performance.

Identification of the psychophysiological and neural substrates of the preparatory processes, and exploration of the ongoing process with higher temporal resolution before and after target detection, could trigger a breakthrough in uncovering the mechanism of PM retrieval. To realize this idea, the combined method of psychophysiological and electrophysiological concurrent recordings would be helpful. Several studies on PM retrieval have reported consistent associations between several ERP components, reflecting the realization of delayed intentions in event-based PM tasks [62–65]. It would be more advantageous to apply the method of heartbeat-evoked brain potentials to understand the mechanisms by which afferent signals from the body have an impact on PM retrieval [66–68]. These multifaceted strategies will enhance our understanding of the underlying psychophysiological and neural mechanisms of PM retrieval.

## Data accessibility

All data are available from the corresponding author upon request.

## References

[1] Einstein GO, McDaniel MA 1990 Normal aging and prospective memory. J. Exp. Psychol. Learn. Mem. Cogn. 16, 717–726. (10.1037/0278-7393.16.4.717) 2142956

[2] Einstein GO, McDaniel MA, Richardson SL, Guynn MJ, Cunfer AR 1995 Aging and prospective memory: examining the influences of self-initiated retrieval processes. J. Exp. Psychol. Learn. Mem. Cogn. 21, 996–1007. (10.1037/0278-7393.21.4.996) 7673871

[3] Brandimonte MA, Einstein GO, McDaniel MA 1996 Prospective memory: theory and applications. Mahwah, NJ: Erlbaum.

[4] Smith RE 2003 The cost of remembering to remember in event-based prospective memory: investigating the capacity demands of delayed intention performance. J. Exp. Psychol. Learn. Mem. Cogn. 29, 347–361. (10.1037/0278-7393.29.3.347) 12776746

[5] Smith RE 2010 What costs do reveal and moving beyond the cost debate: reply to Einstein and McDaniel. J. Exp. Psychol. Learn. Mem. Cogn. 36, 1089–1095. (10.1037/a0019183) 20852726 PMC2940056

[6] Smith RE, Bayen UJ 2004 A multinomial model of event-based prospective memory. J. Exp. Psychol. Learn. Mem. Cogn. 30, 756–777. (10.1037/0278-7393.30.4.756) 15238021

[7] Smith RE, Bayen UJ 2005 The effects of working memory resource availability on prospective memory: a formal modeling approach. Exp. Psychol. 52, 243–256. (10.1027/1618-3169.52.4.243) 16304724

[8] Smith RE, Hunt RR, McVay JC, McConnell MD 2007 The cost of event-based prospective memory: salient target events. J. Exp. Psychol. Learn. Mem. Cogn. 33, 734–746. (10.1037/0278-7393.33.4.734) 17576150

[9] Einstein GO, McDaniel MA 2010 Prospective memory and what costs do not reveal about retrieval processes: a commentary on Smith, Hunt, McVay, and McConnell (2007). J. Exp. Psychol. Learn. Mem. Cogn. 36, 1082–1088; discussion 1089–1095 (10.1037/a0019184) 20565226

[10] Cockburn J 1995 Task interruption in prospective memory: a frontal lobe function? Cortex 31, 87–97. (10.1016/S0010-9452(13)80107-4) 7781322

[11] Cona G, Bisiacchi PS, Sartori G, Scarpazza C 2016 Effects of cue focality on the neural mechanisms of prospective memory: a meta-analysis of neuroimaging studies. Sci. Rep. 6, 25983 (10.1038/srep25983) 27185531 PMC4868976

[12] Cona G, Scarpazza C, Sartori G, Moscovitch M, Bisiacchi PS 2015 Neural bases of prospective memory: a meta-analysis and the ‘Attention to delayed intention’ (AtoDI) model. Neurosci. Biobehav. Rev. 52, 21–37. (10.1016/j.neubiorev.2015.02.007) 25704073

[13] Shallice T, Burgess PW 1991 Deficits in strategy application following frontal lobe damage in man. Brain 114, 727–741. (10.1093/brain/114.2.727) 2043945

[14] Umeda S, Kurosaki Y, Terasawa Y, Kato M, Miyahara Y 2011 Deficits in prospective memory following damage to the prefrontal cortex. Neuropsychologia 49, 2178–2184. (10.1016/j.neuropsychologia.2011.03.036) 21477605

[15] Umeda S, Nagumo Y, Kato M 2006 Dissociative contributions of medial temporal and frontal regions to prospective remembering. Rev. Neurosci. 17, 267–278. (10.1515/REVNEURO.2006.17.1-2.267) 16703957

[16] Bisiacchi PS, Schiff S, Ciccola A, Kliegel M 2009 The role of dual-task and task-switch in prospective memory: behavioural data and neural correlates. Neuropsychologia 47, 1362–1373. (10.1016/j.neuropsychologia.2009.01.034) 19428400

[17] West R 2007 The influence of strategic monitoring on the neural correlates of prospective memory. Mem. Cognit. 35, 1034–1046. (10.3758/BF03193476) 17910187

[18] West R, Herndon RW, Crewdson SJ 2001 Neural activity associated with the realization of a delayed intention. Brain Res. Cogn. Brain Res. 12, 1–9. (10.1016/S0926-6410(01)00014-3) 11489603

[19] West R, Krompinger J 2005 Neural correlates of prospective and retrospective memory. Neuropsychologia 43, 418–433. (10.1016/j.neuropsychologia.2004.06.012) 15707617

[20] Jennings JR, van der Molen MW, Somsen RJ, Terezis C 1990 On the shift from anticipatory heart rate deceleration to acceleratory recovery: revisiting the role of response factors. Psychophysiology 27, 385–395. (10.1111/j.1469-8986.1990.tb02332.x) 2236441

[21] Jennings JR, Vandermolen MW, Brock K, Somsen RJM 1991 Response-inhibition initiates cardiac deceleration—evidence from a sensory-motor compatibility paradigm. Psychophysiology 28, 72–85. (10.1111/j.1469-8986.1991.tb03390.x) 1886965

[22] Schell AM, Catania J 1975 The relationship between cardiac activity and sensory acuity. Psychophysiology 12, 147–151. (10.1111/j.1469-8986.1975.tb01265.x) 1135346

[23] Thayer JF, Lane RD 2000 A model of neurovisceral integration in emotion regulation and dysregulation. J. Affect. Disord. 61, 201–216. (10.1016/S0165-0327(00)00338-4) 11163422

[24] Lewin K, Lippitt R, White R 1939 Patterns of aggressive behavior in experimentally created ‘social climates’. J. Soc. Psychol. 10, 269–299. (10.1080/00224545.1939.9713366)

[25] Zeigarnik B 1927 Über das Behalten von erledigten und unerledigten Handlungen. Psychol. Forsch. 9, 1–85. (10.1007/BF02409755)

[26] Sokolov EN 1963 Higher nervous functions; the orienting reflex. Annu. Rev. Physiol. 25, 545–580. (10.1146/annurev.ph.25.030163.002553) 13977960

[27] Wenzlaff RM, Wegner DM 2000 Thought suppression. Annu. Rev. Psychol. 51, 59–91. (10.1146/annurev.psych.51.1.59) 10751965

[28] Kliegel M, Guynn MJ, Zimmer H 2007 The role of noticing in prospective memory forgetting. Int. J. Psychophysiol. 64, 226–232. (10.1016/j.ijpsycho.2006.09.007) 17113673

[29] Rothen N, Meier B 2014 Psychophysiology of prospective memory. Memory 22, 867–880. (10.1080/09658211.2013.847106) 24138288

[30] Damasio AR 1994 Descartes’ *error: emotion, rationality and the human brain* New York, NY: Putnam.

[31] Damasio AR 1996 The somatic marker hypothesis and the possible functions of the prefrontal cortex. Phil. Trans. R. Soc. Lond. B 351, 1413–1420. (10.1098/rstb.1996.0125) 8941953

[32] Koriat A, Levy-Sadot R 2001 The combined contributions of the cue-familiarity and accessibility heuristics to feelings of knowing. J. Exp. Psychol. Learn. 27, 34–53. (10.1037/0278-7393.27.1.34) 11204106

[33] Reder LM 1987 Strategy selection in question answering. Cogn. Psychol. 19, 90–138. (10.1016/0010-0285(87)90005-3)

[34] Arana JM, Meilan JJ, Perez E 2008 The effect of personality variables in the prediction of the execution of different prospective memory tasks in the laboratory. Scand. J. Psychol. 49, 403–411. (10.1111/j.1467-9450.2008.00671.x) 18705674

[35] Cuttler C, Graf P 2007 Personality predicts prospective memory task performance: an adult lifespan study. Scand. J. Psychol. 48, 215–231. (10.1111/j.1467-9450.2007.00570.x) 17518914

[36] Pearman A, Storandt M 2005 Self-discipline and self-consciousness predict subjective memory in older adults. J. Gerontol. B. Psychol. Sci. Soc. Sci. 60, P153–P157. (10.1093/geronb/60.3.P153) 15860785

[37] Salthouse TA, Berish DE, Siedlecki KL 2004 Construct validity and age sensitivity of prospective memory. Mem. Cognit. 32, 1133–1148. (10.3758/BF03196887) 15813495

[38] Arnold NR, Bayen UJ, Bohm MF 2015 Is prospective memory related to depression and anxiety? A hierarchical MPT modelling approach. Memory 23, 1215–1228. (10.1080/09658211.2014.969276) 25337864

[39] Cameron OG 2001 Interoception: the inside story---a model for psychosomatic processes. Psychosom. Med. 63, 697–710. (10.1097/00006842-200109000-00001) 11573016

[40] Sherrington CS 1906 The integrative action of the nervous system. New Haven, CT: Yale University Press.

[41] Garfinkel SN, Seth AK, Barrett AB, Suzuki K, Critchley HD 2015 Knowing your own heart: distinguishing interoceptive accuracy from interoceptive awareness. Biol. Psychol. 104, 65–74. (10.1016/j.biopsycho.2014.11.004) 25451381

[42] Critchley HD, Wiens S, Rotshtein P, Ohman A, Dolan RJ 2004 Neural systems supporting interoceptive awareness. Nat. Neurosci. 7, 189–195. (10.1038/nn1176) 14730305

[43] Terasawa Y, Moriguchi Y, Tochizawa S, Umeda S 2014 Interoceptive sensitivity predicts sensitivity to the emotions of others. Cogn. Emotion 28, 1435–1448. (10.1080/02699931.2014.888988) 24559130

[44] Schandry R 1981 Heart beat perception and emotional experience. Psychophysiology 18, 483–488. (10.1111/j.1469-8986.1981.tb02486.x) 7267933

[45] Barrett LF, Quigley KS, Bliss-Moreau E, Aronson KR 2004 Interoceptive sensitivity and self-reports of emotional experience. J. Pers. Soc. Psychol. 87, 684–697. (10.1037/0022-3514.87.5.684) 15535779 PMC1224728

[46] Pollatos O, Kirsch W, Schandry R 2005 Brain structures involved in interoceptive awareness and cardioafferent signal processing: a dipole source localization study. Hum. Brain Mapp. 26, 54–64. (10.1002/hbm.20121) 15852466 PMC6871699

[47] Kane MJ, Conway AR, Miura TK, Colflesh GJ 2007 Working memory, attention control, and the N-back task: a question of construct validity. J. Exp. Psychol. Learn. Mem. Cogn. 33, 615–622. (10.1037/0278-7393.33.3.615) 17470009

[48] Ehlers A, Breuer P 1992 Increased cardiac awareness in panic disorder. J. Abnorm. Psychol. 101, 371–382. (10.1037/0021-843X.101.3.371) 1500594

[49] Pollatos O, Herbert BM, Kaufmann C, Auer DP, Schandry R 2007 Interoceptive awareness, anxiety and cardiovascular reactivity to isometric exercise. Int. J. Psychophysiol. 65, 167–173. (10.1016/j.ijpsycho.2007.03.005) 17449123

[50] Pollatos O, Schandry R 2008 Emotional processing and emotional memory are modulated by interoceptive awareness. Cogn. Emotion 22, 272–287. (10.1080/02699930701357535)

[51] Pollatos O, Traut-Mattausch E, Schandry R 2009 Differential effects of anxiety and depression on interoceptive accuracy. Depress. Anxiety 26, 167–173. (10.1002/da.20504) 19152366

[52] Pollatos O, Traut-Mattausch E, Schroeder H, Schandry R 2007 Interoceptive awareness mediates the relationship between anxiety and the intensity of unpleasant feelings. J. Anxiety Disord. 21, 931–943. (10.1016/j.janxdis.2006.12.004) 17257810

[53] Dunn BD, Galton HC, Morgan R, Evans D, Oliver C, Meyer M, Cusack R, Lawrence AD, Dalgleish T 2010 Listening to your heart. How interoception shapes emotion experience and intuitive decision making. Psychol. Sci. 21, 1835–1844. (10.1177/0956797610389191) 21106893

[54] West R, Craik FI 1999 Age-related decline in prospective memory: the roles of cue accessibility and cue sensitivity. Psychol. Aging 14, 264–272. (10.1037/0882-7974.14.2.264) 10403713

[55] Burgess PW, Scott SK, Frith CD 2003 The role of the rostral frontal cortex (area 10) in prospective memory: a lateral versus medial dissociation. Neuropsychologia 41, 906–918. (10.1016/S0028-3932(02)00327-5) 12667527

[56] McDaniel MA, Guynn MJ, Einstein GO, Breneiser J 2004 Cue-focused and reflexive-associative processes in prospective memory retrieval. J. Exp. Psychol. Learn. Mem. Cogn. 30, 605–614. (10.1037/0278-7393.30.3.605) 15099129

[57] Einstein GO, McDaniel MA, Thomas R, Mayfield S, Shank H, Morrisette N, Breneiser J 2005 Multiple processes in prospective memory retrieval: factors determining monitoring versus spontaneous retrieval. J. Exp. Psychol. Gen. 134, 327–342. (10.1037/0096-3445.134.3.327) 16131267

[58] McDaniel MA, Einstein GO 2000 Strategic and automatic processes in prospective memory retrieval: a multiprocess framework. Appl. Cogn. Psychol. 14, S127–S144. (10.1002/acp.775)

[59] Einstein GO, McDaniel MA 2005 Prospective memory: multiple retrieval processes. Curr. Dir. Psychol. Sci. 14, 286–290. (10.1111/j.0963-7214.2005.00382.x)

[60] Scullin MK, McDaniel MA, Shelton JT, Lee JH 2010 Focal/nonfocal cue effects in prospective memory: monitoring difficulty or different retrieval processes? J. Exp. Psychol. Learn 36, 736–749. (10.1037/a0018971) PMC2864946 20438269

[61] McDaniel MA, Shelton JT, Breneiser JE, Moynan S, Balota DA 2011 Focal and nonfocal prospective memory performance in very mild dementia: a signature decline. Neuropsychology 25, 387–396. (10.1037/a0021682) 21443344 PMC3086982

[62] West R 2011 The temporal dynamics of prospective memory: a review of the ERP and prospective memory literature. Neuropsychologia 49, 2233–2245. (10.1016/j.neuropsychologia.2010.12.028) 21187107

[63] West R, Bowry R 2005 Effects of aging and working memory demands on prospective memory. Psychophysiology 42, 698–712. (10.1111/j.1469-8986.2005.00361.x) 16364065

[64] West R, Bowry R, Krompinger J 2006 The effects of working memory demands on the neural correlates of prospective memory. Neuropsychologia 44, 197–207. (10.1016/j.neuropsychologia.2005.05.003) 15936045

[65] West R, McNerney MW, Travers S 2007 Gone but not forgotten: the effects of cancelled intentions on the neural correlates of prospective memory. Int. J. Psychophysiol. 64, 215–225. (10.1016/j.ijpsycho.2006.09.004) 17107728

[66] Fukushima H, Terasawa Y, Umeda S 2011 Association between interoception and empathy: evidence from heartbeat-evoked brain potential. Int. J. Psychophysiol. 79, 259–265. (10.1016/j.ijpsycho.2010.10.015) 21055427

[67] Pollatos O, Schandry R 2004 Accuracy of heartbeat perception is reflected in the amplitude of the heartbeat-evoked brain potential. Psychophysiology 41, 476–482. (10.1111/1469-8986.2004.00170.x) 15102134

[68] Schandry R, Sparrer B, Weitkunat R 1986 From the heart to the brain: a study of heartbeat contingent scalp potentials. Int. J. Neurosci. 30, 261–275. (10.3109/00207458608985677) 3793380

